# Associations of Serum Niacin and Nicotinamide with Child Behavior: A Longitudinal Study in China

**DOI:** 10.3390/nu18203344

**Published:** 2026-10-12

**Authors:** Xinhan Zhang, Kuiqing Huang, Chen Liu, Yi Wang, Jinghan Jiang, Zhijian Chen, Jie Xiang, Peiwei Xu

**Affiliations:** 1 Zhejiang Provincial Center for Disease Control and Prevention, Hangzhou 310051, China; 2 Yiwu Center for Disease Control and Prevention, Yiwu 322000, China

**Keywords:** niacin, nicotinamide, vitamin B3, NAD+, Child Behavior Checklist, CBCL, child behavior, nutrition, children

## Abstract

**Background/Objectives**: Nicotinic acid (NA; niacin) and nicotinamide (NAM) participate in nicotinamide adenine dinucleotide metabolism, but pediatric evidence relating serum concentrations of these vitamers to behavioral-problem scores is limited. **Methods**: We conducted an exploratory analysis of children assessed in Yiwu, Zhejiang Province, China, with serum NA and NAM concentrations recorded in 2021 and 2025 and caregiver-reported Child Behavior Checklist (CBCL) outcomes in 2025. Vitamin B3 was measured by high-performance liquid chromatography–tandem mass spectrometry (HPLC–MS/MS). Internalizing, externalizing, and total-problem raw scores were the broadband outcomes; seven common raw subscales were exploratory outcomes. Multivariable linear models and three-knot restricted cubic splines (RCSs) were conducted to assess the associations between vitamin B3 and CBCL scores. Benjamini–Hochberg correction and exposure-by-sex interaction tests were used. **Results**: Pooled associations were compatible with the null after correction. Per interquartile-range (IQR) increase in 2021 NA, boys’ adjusted contrasts were −2.18 (95% CI, −4.22 to −0.13) for internalizing, −3.04 (−5.05 to −1.04) for externalizing, and −5.33 (−9.18 to −1.47) for total problems. In sex-stratified RCS analyses, 2021 NA was associated with externalizing (q-overall = 0.040) and total problems (q-overall = 0.023) in boys, whereas 2025 NA was associated with these outcomes in girls (both q-overall = 0.042). Sex-stratified two-degree-of-freedom RCS models had limited sensitivity for small-to-modest associations. **Conclusions**: The sex-interaction and sex-stratified RCS patterns for NA are hypothesis-generating and require independent, prespecified replication.

## 1. Introduction

Childhood behavioral and emotional problems are a major public health concern. A worldwide meta-analysis estimated that mental disorders affect 13.4% of children and adolescents [1]. The Child Behavior Checklist (CBCL), part of the Achenbach System of Empirically Based Assessment, is widely used to assess caregiver-reported behavioral and emotional problems in children [2,3]. CBCL broadband scores, including internalizing, externalizing, and total-problem scores, provide a structured approach for summarizing behavioral symptoms across domains. Externalizing and attention-related symptoms are common reasons for referral to child mental health services [4], and early self-regulation difficulties may predict later health and social outcomes [5].

Identifying modifiable biological correlates may clarify opportunities for prevention. In China, a national CBCL survey of children aged 6–16 years reported substantial behavioral and emotional morbidity and variation by age and sex [6]. More broadly, systematic reviews and prospective studies suggest that diet quality is associated with child and adolescent mental health, although residual confounding and bidirectionality remain important concerns [7,8,9,10,11]. Previous studies have mostly focused on overall diet quality, dietary patterns, or broad nutrient groups rather than measured vitamin metabolites. As a result, evidence remains limited for specific nutrients or vitamers.

Nicotinic acid (NA; niacin) and nicotinamide (NAM), two vitamin B3 vitamers, are distinct nicotinamide adenine dinucleotide (NAD+) precursors with tissue-specific metabolic routes [12]. They contribute to pools of NAD+ that support cellular redox reactions, energy production, DNA repair, and mitochondrial homeostasis [10,13]. Isotope-tracer work further shows that circulating precursor availability, tissue NAD concentration, and NAD turnover are not interchangeable measures [14]. These pathways are relevant to neuronal function, although much of the evidence derives from mechanistic work or adults [10,15,16].

Vitamin B3 is obtained as NA and NAM from foods including meat, fish, poultry, legumes, grains, and fortified foods; NAM can also be generated endogenously from dietary tryptophan [12]. Circulating NA and NAM may therefore vary with recent dietary intake, supplement use, tryptophan metabolism, overall diet quality, tissue utilization, inflammation, and renal or hepatic function. Serum concentrations cannot be equated with dietary intake or intracellular NAD status. The specific evidence gap is the absence of longitudinal pediatric studies relating measured NA and NAM concentrations, rather than intake estimates alone, to standardized caregiver-reported behavioral outcomes.

Direct pediatric evidence on vitamin B3 and behavior remains sparse. A cross-sectional study in Korean children and adolescents linked dietary niacin with attention-related cognitive performance, but did not assess broad behavioral problems [17]. Adult observational and clinical studies have also linked niacin intake or NAD+ precursor supplementation with cognitive or neurological outcomes, although these findings cannot be directly generalized to children [18,19]. Thus, the existing literature supports a plausible link between vitamin B3-related metabolism and neurobehavioral function, but evidence connecting measured NA or NAM concentrations with CBCL-assessed behavioral problems in children remains limited.

We therefore examined whether serum NA and NAM concentrations measured in 2021 and 2025 were associated with internalizing, externalizing, and total-problem raw scores in 2025. The 2021 analyses were temporally prospective and the 2025 analyses were cross-sectional; both were observational. We also evaluated sex interactions and nonlinear exposure–response patterns as exploratory analyses.

## 2. Materials and Methods

### 2.1. Study Design and Participants

This observational analysis was nested within a school-based prospective cohort in Yiwu, Zhejiang Province, China. The 2021 baseline survey enrolled 294 children aged 7–10 years (Figure 1). In the analytic source workbook, 250 records had biomarker or covariate data available. Matching nonmissing participant identifiers between the main and CBCL worksheets yielded 222 linked children (119 boys and 103 girls); 206 had all three broadband CBCL outcomes recorded in 2025. Thus, 294 denotes the baseline cohort, 250 the available analytic source records, 222 the linked records, and 201–205 the model-specific complete-case samples. The 2021 exposure analyses were temporally prospective with respect to 2025 outcomes, whereas the 2025 exposure analyses were cross-sectional. Written informed consent was obtained from all participants and/or their legal guardians. The study protocol was approved by the Ethics and Human Subject Committee of the Zhejiang Provincial Center for Disease Control and Prevention (2020-040-01 and 2025-022-01). Serum was transferred to 1.5 mL polypropylene tubes and stored at −80 °C until analysis.

### 2.2. Serum Nicotinic Acid and Nicotinamide

Serum vitamin B3 was assessed as nicotinic acid (NA; niacin) and nicotinamide (NAM). High-performance liquid chromatography–tandem mass spectrometry (HPLC-MS/MS) was used for vitamin B3 and other water-soluble B vitamins.

Serum vitamin B3 was quantified as NA and NAM. An HPLC-MS/MS system (AB Sciex TRIPLE QUAD 5500) was used to measure vitamin B3 as part of a water-soluble B-vitamin panel [20]. Values were natural-log transformed because their distributions were right-skewed.

### 2.3. Child Behavior Measurement

The Child Behavior Checklist (CBCL), part of the Achenbach System of Empirically Based Assessment, is widely used to assess caregiver-reported behavioral and emotional problems in children [5,6]. Caregiver-reported CBCL raw scores were used as behavioral outcomes. The primary broadband outcomes were internalizing, externalizing, and total-problem raw scores. Exploratory outcomes included depression, compulsive activity, somatic complaints, social withdrawal, hyperactivity, aggressive behavior, and delinquent behavior.

### 2.4. Statistical Analysis

Participant characteristics were summarized using means and standard deviations or medians and interquartile ranges (IQRs). Multivariable linear regression models were used to estimate associations between each vitamin B3 and each CBCL outcome. Models were adjusted for age, sex, passive smoking, body mass index (BMI, kg/m^2^), and residence duration. Sex-stratified contrasts were estimated through exposure-by-sex interaction models. Primary analyses evaluated pooled associations with internalizing, externalizing, and total-problem raw scores. Secondary analyses evaluated selected CBCL subscales. Restricted cubic spline models with three knots were used to explore possible nonlinear associations for broadband outcomes [21]. Benjamini–Hochberg false-discovery-rate correction was applied within prespecified families of tests [22].

We conducted a model-based minimum detectable effect-size (MDES) sensitivity analysis. For each model-specific complete-case sample, we estimated the partial R^2^ detectable with 80% power using noncentral F distributions at a two-sided α level of 0.05. We also evaluated α = 0.05/12 as a conservative fixed sensitivity bound for the 12 primary tests. One-degree-of-freedom tests were used for pooled linear associations and exposure-by-sex interactions, and two-degree-of-freedom tests for overall RCS associations. At 80% power and α = 0.05, pooled linear associations and exposure-by-sex interactions (*n* = 201–205) could detect partial R^2^ values of 0.038–0.039. The pooled two-degree-of-freedom RCS tests could detect partial R^2^ values of 0.047–0.048. In contrast, sex-stratified two-degree-of-freedom RCS tests required partial R^2^ values of 0.089–0.090 among boys (*n* = 104–106) and 0.095–0.097 among girls (*n* = 97–99). Under the conservative α = 0.05/12 threshold, the corresponding values were 0.066–0.067 for pooled linear and interaction analyses, 0.076–0.078 for pooled RCS analyses, 0.143–0.146 among boys, and 0.153–0.156 among girls. Thus, the sex-stratified spline analyses had limited sensitivity for small-to-modest associations.

## 3. Results

### 3.1. Participant Flow and Characteristics

Of the 294 children enrolled at baseline, 250 records were available in the analytic source workbook. Matching to the CBCL worksheet produced 222 linked children (119 boys and 103 girls); the three broadband raw scores were available for 206 children. The mean age was 8.13 ± 0.37 years. Boys had mean internalizing, externalizing, and total-problem raw scores of 4.89, 5.67, and 10.65, respectively (Table 1). The corresponding values in girls were 5.05, 5.62, and 10.97. The median concentrations of NA and NAM was 0.29 (0.21, 0.38) μmol/L and 16.70 (12.62, 20.33) μmol/L at baseline, and was 0.61 (0.36, 0.91) μmol/L and 23.84 (17.41, 32.08) μmol/L at follow-up.

### 3.2. Associations of NA and NAM with Broadband CBCL Scores

In the overall sample, neither NA nor NAM measured at baseline or follow-up was associated with internalizing, externalizing, or total-problem scores (Figure 2). Sex-stratified analyses revealed distinct patterns. Among boys, higher NA concentrations at baseline were associated with lower internalizing (β  =  −3.733, 95% CI: −6.686 to −0.779), externalizing (β  =  −5.204, 95% CI: −8.517 to −1.891), and total-problem scores (β  =  −9.198, 95% CI: −14.632 to −3.763). The associations with externalizing and total-problem T scores remained significant after FDR correction, whereas the association with internalizing symptoms did not. No significant associations were observed for NAM at either time point. Among girls, higher nicotinic acid concentrations at follow-up were nominally associated with higher externalizing (β  =  2.240, 95% CI: 0.180 to 4.300) and total-problem scores (β  =  4.248, 95% CI: 0.601–7.894); however, neither association remained significant after Benjamini–Hochberg false-discovery-rate correction (FDR-adjusted *p*  =  0.24 and 0.12, respectively).

### 3.3. Associations of NA and NAM with Subscale Outcomes

Serum NA levels of boys at baseline were inversely associated with somatic complaints (β = −0.31, 95% CI: −0.50 to −0.11; q-interaction = 0.009), compulsive activity (β = −0.49, −0.93 to −0.05; q-interaction = 0.024), delinquent behavior (β = −0.54, −0.86 to −0.23; q-interaction = 0.024), and aggressive behavior (β = −1.77, −3.04 to −0.50; q-interaction = 0.042) scores at follow-up in Figure 3. The corresponding girls’ contrasts were inverse or nonsignificant. The associations between NA and depression at follow-up were positive in girls (β = 1.06, 0.23 to 1.88) and null in boys (β = −0.49, −0.98 to 0.004; q-interaction = 0.023).

### 3.4. Restricted Cubic Spline Analyses

In pooled RCS models (Figure S1), no overall spline test survived correction across 12 tests (q-overall range, 0.623–0.993), and no test showed evidence of departure from linearity after correction (all q-nonlinear = 0.939). Sex-stratified RCS models identified differed patterns for NA, but not NAM (Figure 3). Among boys, NA at baseline was associated with externalizing (*p*-overall = 0.0067; q-overall = 0.040) and total-problem scores (*p*-overall = 0.0019; q-overall = 0.023) in Figure 4. Among girls, NA was associated with externalizing (*p*-overall = 0.0070; q-overall = 0.042) and total-problem scores (*p*-overall = 0.0057; q-overall = 0.042) at follow-up. The curves were broadly monotonic around the reference median (Figure S2).

## 4. Discussion

In this exploratory observational analysis, pooled associations of serum NA and NAM with broadband and common-subscale CBCL raw scores were compatible with the null after correction for multiple testing. Interaction analyses suggested sex heterogeneity for 2021 NA across the three broadband outcomes and several low-count subscales. The sex-stratified RCS results were directionally coherent with the interaction contrasts, but neither pooled nor sex-stratified models showed corrected evidence of nonlinearity.

The 2021 NA pattern in boys is sufficiently coherent to motivate replication, but it does not establish a protective effect. Pooled associations were null, the interaction analyses were exploratory, and raw-score interactions can be sensitive to scale, distribution, exposure overlap, and influential observations. Candidate modifiers beyond sex include dietary intake and supplement use, tryptophan metabolism, overall B-vitamin status, pubertal timing, body composition, inflammation, renal or hepatic function, socioeconomic conditions, family context, and caregiver reporting. Independent replication should prespecify the interaction family and measure these factors.

The inverse associations observed in boys are biologically plausible in the context of vitamin B3 and NAD+ biology. NA and NAM contribute to NAD+ metabolism, which is involved in cellular energy production, redox balance, DNA repair, stress responses, and neuronal function [12,13,15,16,23]. These processes are relevant during childhood because the developing nervous system is sensitive to nutritional and metabolic conditions. The findings are also directionally consistent with a pediatric study in which higher dietary niacin intake was associated with fewer omission errors in attention-related cognitive testing [17]. Nevertheless, omission errors are not equivalent to CBCL scores, and dietary intake is not equivalent to serum vitamin B3 vitamer concentration.

However, the observed sex interactions should not be interpreted as evidence that NA harms girls. The analyses were data-informed, the pooled associations were null, and raw-score interactions can be sensitive to scale, distribution, exposure overlap, and influential observations. Sex-specific diet–behavior associations have also been reported in adolescent cohorts, but their inconsistency reinforces the need for prespecified replication rather than mechanistic interpretation of subgroup findings [9]. The sex-specific pattern could also arise from differences in pubertal timing, body composition, exposure overlap, or behavioral reporting, none of which could be fully addressed in the current analysis. Independent replication with a prespecified interaction family is required.

The results should also be interpreted in relation to the broader diet and mental health literature. Reviews of child and adolescent studies suggest that healthier dietary patterns are often associated with more favorable mental health outcomes, but the evidence remains heterogeneous and vulnerable to residual confounding [7,8]. Our study focused on measured vitamin B3-related analytes rather than diet quality, which may reduce some measurement limitations of dietary questionnaires but introduces other uncertainties related to biological matrix, short-term metabolic variation, and laboratory comparability. Serum NA and NAM may reflect recent intake, metabolism, broader B-vitamin status, health status, or unmeasured socioeconomic and family factors rather than a specific vitamin B3 effect.

This study has several strengths. It used measured NA and NAM rather than dietary intake alone, included two recorded analyte time points, evaluated broadband CBCL outcomes and exploratory subscales, applied multiplicity correction, tested sex interactions formally, and explored nonlinear exposure–response patterns using restricted cubic splines.

The study also has substantial limitations. First, the observational design precludes causal inference and temporal interpretation unless the chronology of analyte and CBCL measurements is verified. Second, raw CBCL scores were used because the available variables could not be reliably converted to validated norm-referenced T scores without additional documentation. Third, the covariate set was limited and lacked a complete causal rationale; residual confounding by diet, socioeconomic conditions, family factors, developmental status, inflammation, and renal or hepatic function remains plausible. Fourth, subscale outcomes were low-count and zero-heavy, making linear model estimates difficult to interpret. Fifth, the MDES analysis indicates that the sex-stratified RCS models were insensitive to small-to-modest partial associations. Accordingly, null spline tests cannot exclude effects below the reported detection thresholds, and the corrected sex-stratified signals should not be interpreted as establishing reproducible sex-specific dose–response relations. Finally, sex interaction analyses were exploratory and require independent validation.

## 5. Conclusions

In conclusion, this exploratory study found no corrected evidence of pooled associations between serum NA or NAM and broadband CBCL raw scores in children. Among boys, higher serum nicotinic acid concentrations were associated with lower CBCL behavioral-problem scores. Future studies should use larger longitudinal cohorts with balanced sex-specific samples; repeated measures of diet, supplements, NA, NAM, and relevant metabolic biomarkers; validated CBCL scoring; prespecified interaction and multiplicity plans; and broader confounder measurement to determine whether these patterns are reproducible.

## Figures and Tables

**Figure 1:**
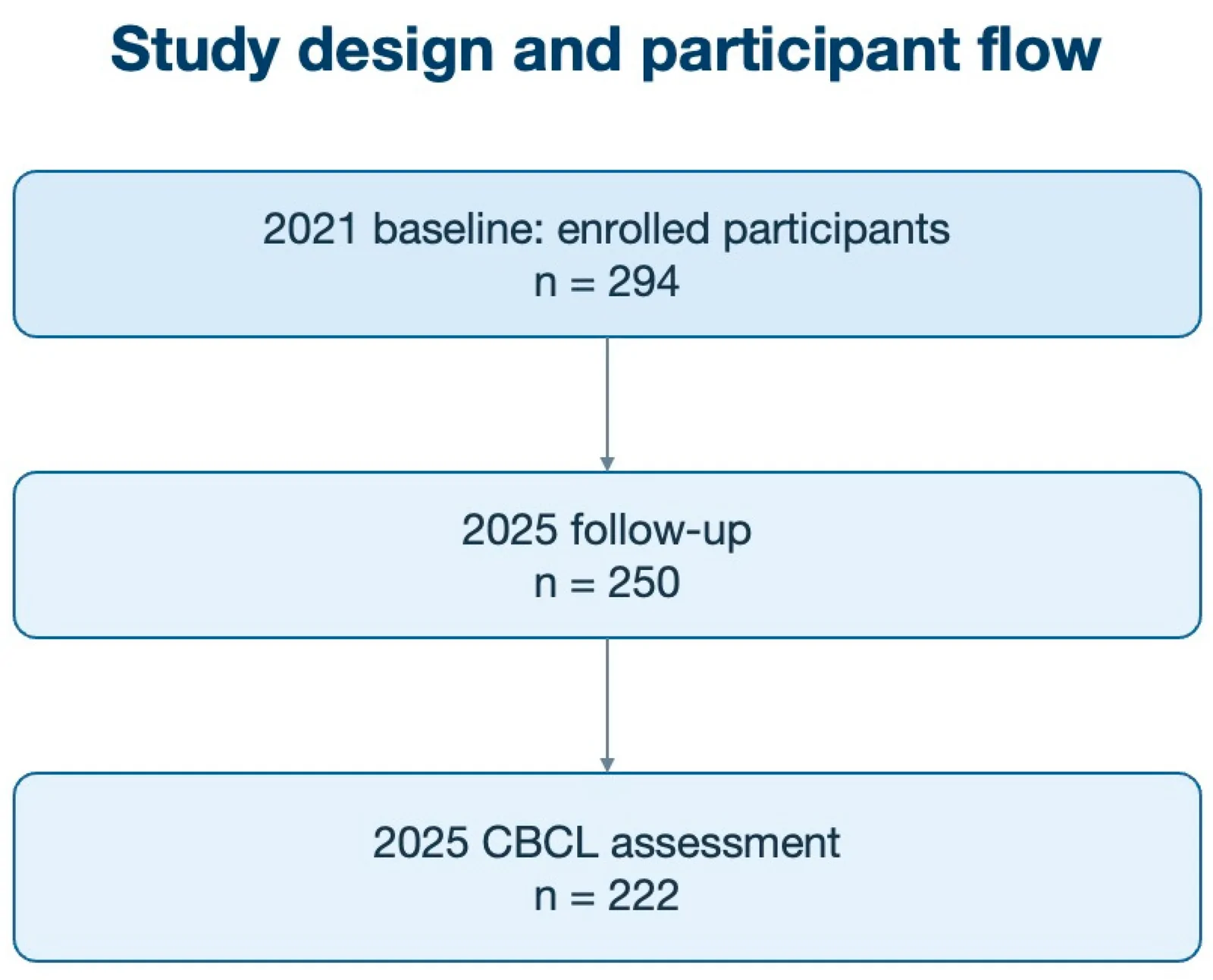
Study design and participant flow. CBCL, Child Behavior Checklist. The figure distinguishes the 2021 baseline cohort (*n* = 294), analytic source records (*n* = 250), linkage to the 2025 CBCL worksheet (*n* = 222), and complete-case samples for the pooled broadband models. The 2021 exposure models are prospective with respect to 2025 CBCL outcomes; the 2025 exposure models are cross-sectional.

**Figure 2:**
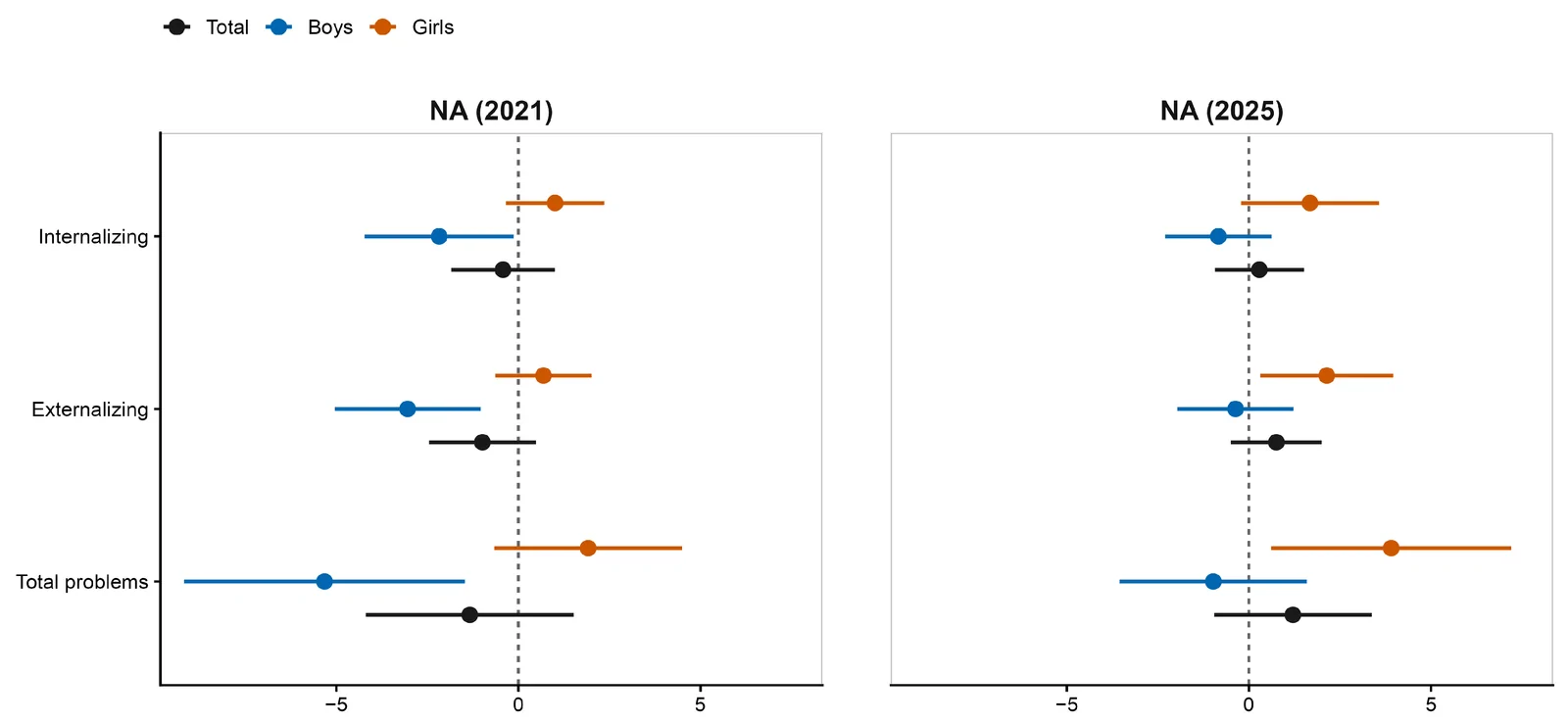
Adjusted associations of serum nicotinic acid with broadband CBCL scores. Points and horizontal lines indicate adjusted estimates and 95% confidence intervals. Color identifies the analysis group only; all points use the same shape. Formal inference is based on exposure-by-sex interaction *p* values and Benjamini–Hochberg-adjusted q values.

**Figure 3:**
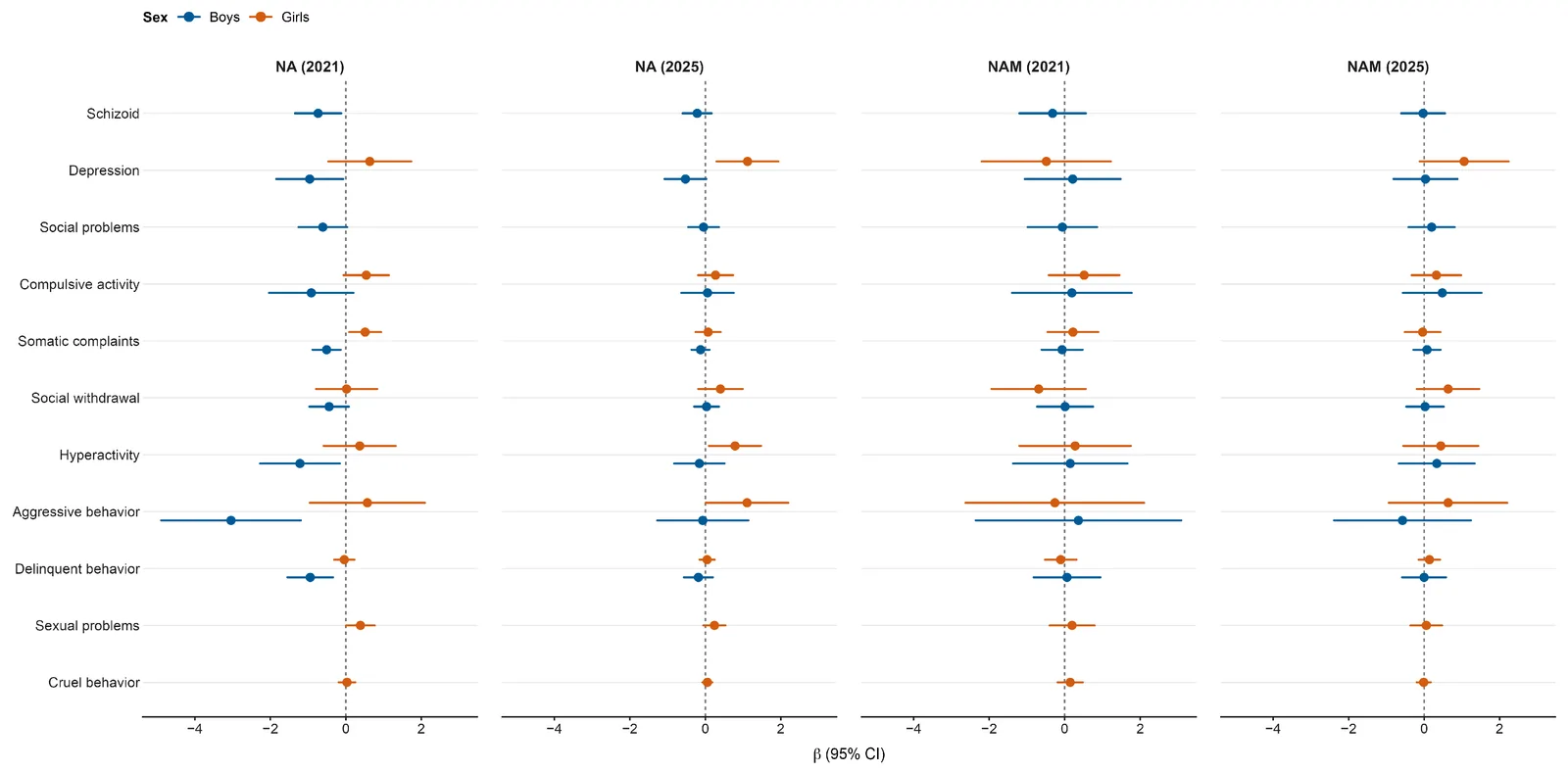
Adjusted associations of serum nicotinic acid and nicotinamide with exploratory CBCL subscale scores. Points and horizontal lines indicate adjusted estimates and 95% confidence intervals. Color identifies sex only; all points use the same shape. Formal inference is based on interaction *p* values and adjusted q values.

**Figure 4:**
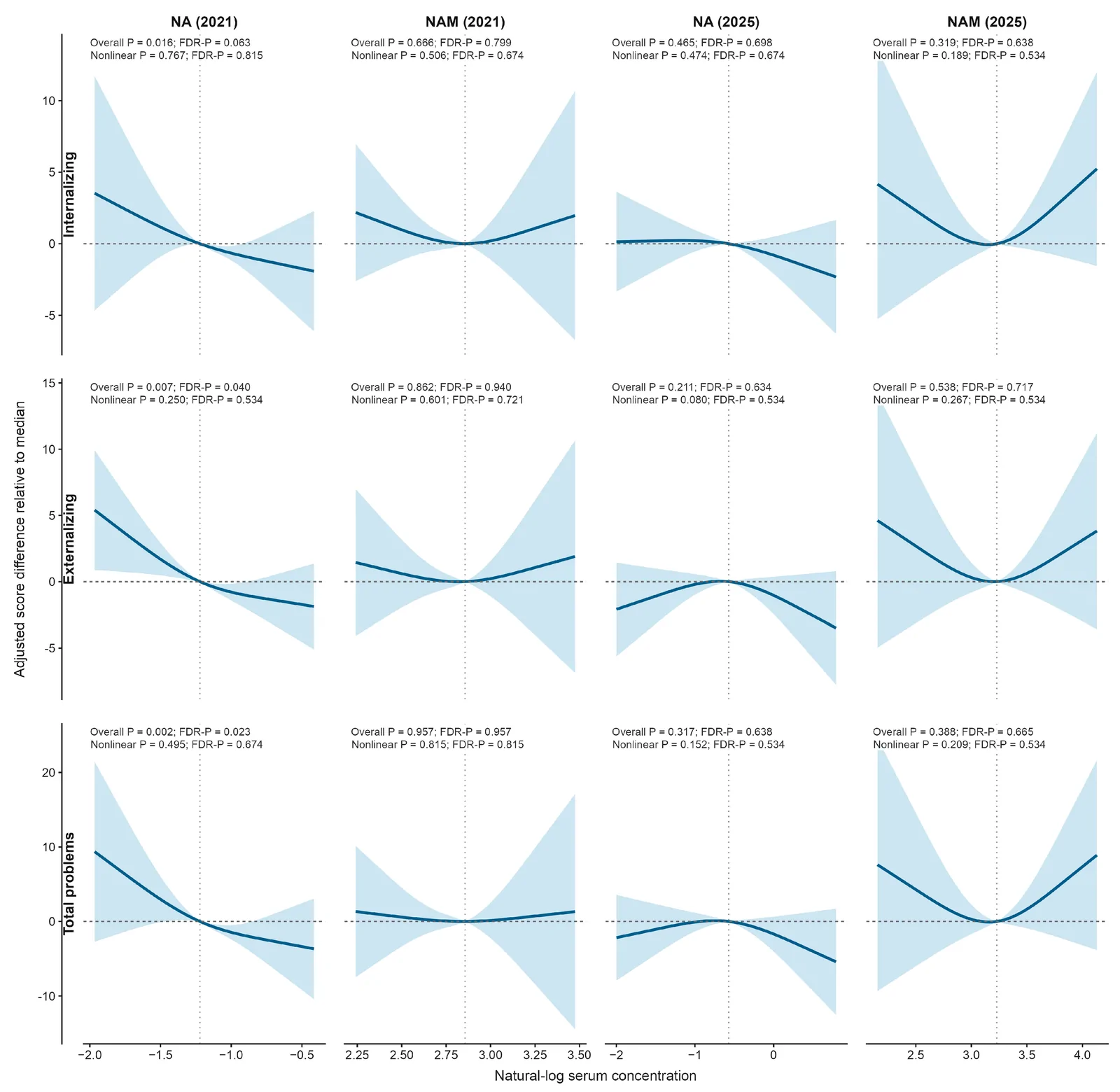
Restricted cubic spline associations of serum niacin and nicotinamide with CBCL outcomes for boys. Solid curves show adjusted score differences relative to the exposure median; shaded bands show 95% confidence intervals, and the vertical dotted line marks the reference median. Panel labels report *p*-overall, *p*-nonlinear, and adjusted q values; apparent curve shape alone is not evidence of nonlinearity.

**Table 1:** Characteristics of the participants.

Characteristics	Overall *n* = 250	Boys *n* = 128	Girls *n* = 111	*p*
Age (years)	8.13 ± 0.37	8.16 ± 0.41	8.10 ± 0.30	0.17
BMI (kg/m^2^)	16.65 ± 2.86	16.79 ± 2.91	16.49 ± 2.81	0.41
Passive smoking (yes, %)	42 (17.6)	25 (19.5)	17 (15.3)	0.49
Schizoid	0.97 ± 1.49	0.97 ± 1.49	/	/
Depression	1.61 ± 2.54	1.19 ± 2.11	2.07 ± 2.87	0.01
Social problems	0.81 ± 1.53	0.81 ± 1.53	/	/
Compulsive activity	1.13 ± 2.21	1.47 ± 2.64	0.76 ± 1.57	0.02
Somatic complaints	0.57 ± 1.03	0.45 ± 0.91	0.70 ± 1.13	0.08
Social withdrawal	1.01 ± 1.77	0.53 ± 1.22	1.53 ± 2.11	<0.001
Hyperactivity	1.69 ± 2.49	1.62 ± 2.51	1.77 ± 2.49	0.67
Aggressive behavior	3.02 +−4.30	3.15 ± 4.50	2.88 ± 4.09	0.65
Delinquent behavior	0.62 ± 1.21	0.91 ± 1.48	0.30 ± 0.71	<0.001
Sexual problem	0.49 ± 1.00	/	0.49 ± 1.00	/
Cruel	0.17 ± 0.59	/	0.17 ± 0.59	/
Internalizing	4.97 ± 6.86	4.89 ± 7.10	5.05 ± 6.61	0.87
Externalizing	5.65 ± 7.82	5.67 ± 8.02	5.62 ± 7.64	0.96
Total	10.81 ± 13.18	10.65 ± 13.33	10.97 ± 13.08	0.86
NA (baseline, μmol/L)	0.61 [0.35, 0.89]	0.64 [0.35, 0.94]	0.61 [0.37, 0.88]	0.95
NAM (baseline, μmol/L)	22.93 [17.34, 31.85]	25.43 [18.29, 33.43]	22.63 [15.24, 31.07]	0.15
NA (follow-up, μmol/L)	0.61 [0.35, 0.89]	0.64 [0.35, 0.94]	0.61 [0.37, 0.88]	0.95
NAM (follow-up, μmol/L)	22.93 [17.34, 31.85]	25.43 [18.29, 33.43]	22.63 [15.24, 31.07]	0.15

Notes: Values are mean ± SD or median (IQR). NA, nicotinic acid; NAM, nicotinamide.

## Data Availability

The data presented in this study are available on request from the corresponding author due to ethical and participant-privacy restrictions.

## Supplementary Materials

The following supporting information can be downloaded at https://www.mdpi.com/article/10.3390/nu18203344/s1, Figure S1. Restricted cubic spline associations of serum niacin and nicotinamide with CBCL outcomes for total participants; Figure S2. Restricted cubic spline associations of serum niacin and nicotinamide with CBCL outcomes for girls.

## Abbreviations

The following abbreviations are used in this manuscript:

NA	Nicotinic
NAM	Nicotinamide
CBCL	Child Behavior Checklist
RCS	Restricted Cubic Splines
NAD+	Nicotinamide Adenine Dinucleotide
IQR	Interquartile Range
95%CI	95% confidence interval
BMI	Body Mass Index
