# Supplementary material for: Associations of Serum Niacin and Nicotinamide with Child Behavior: A Longitudinal Study in China
Source: Nutrients. 2026 Oct 12;18(20):3344. doi: 10.3390/nu18203344 (PMC13708041; doi:10.3390/nu18203344)
Supplement: Supplementary file 1 [file nutrients-18-03344-s001.zip › nutrients-4537323-supplementary.pdf]

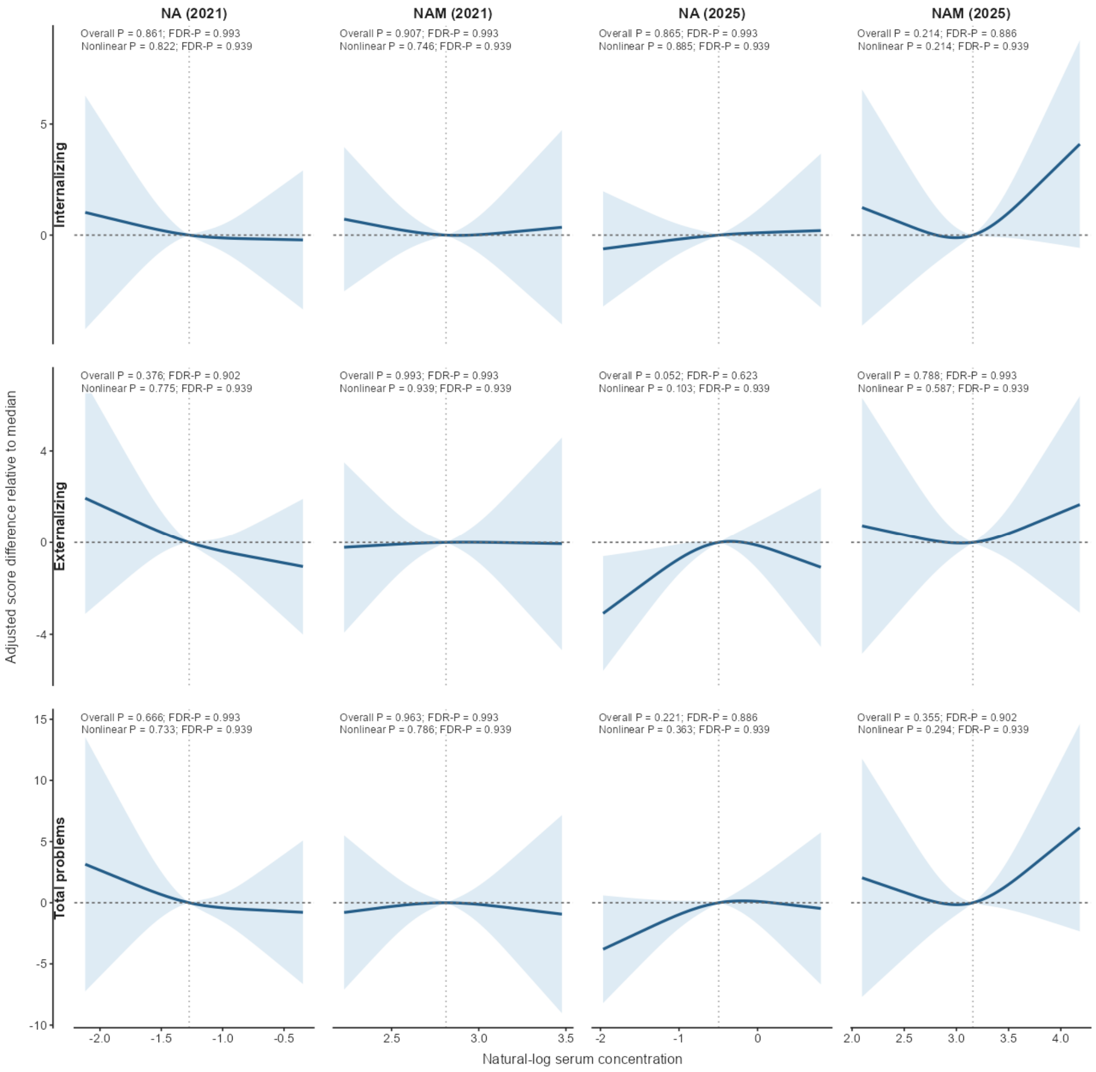

Figure S1. Restricted cubic spline associations of serum niacin and nicotinamide with CBCL outcomes for total participants.

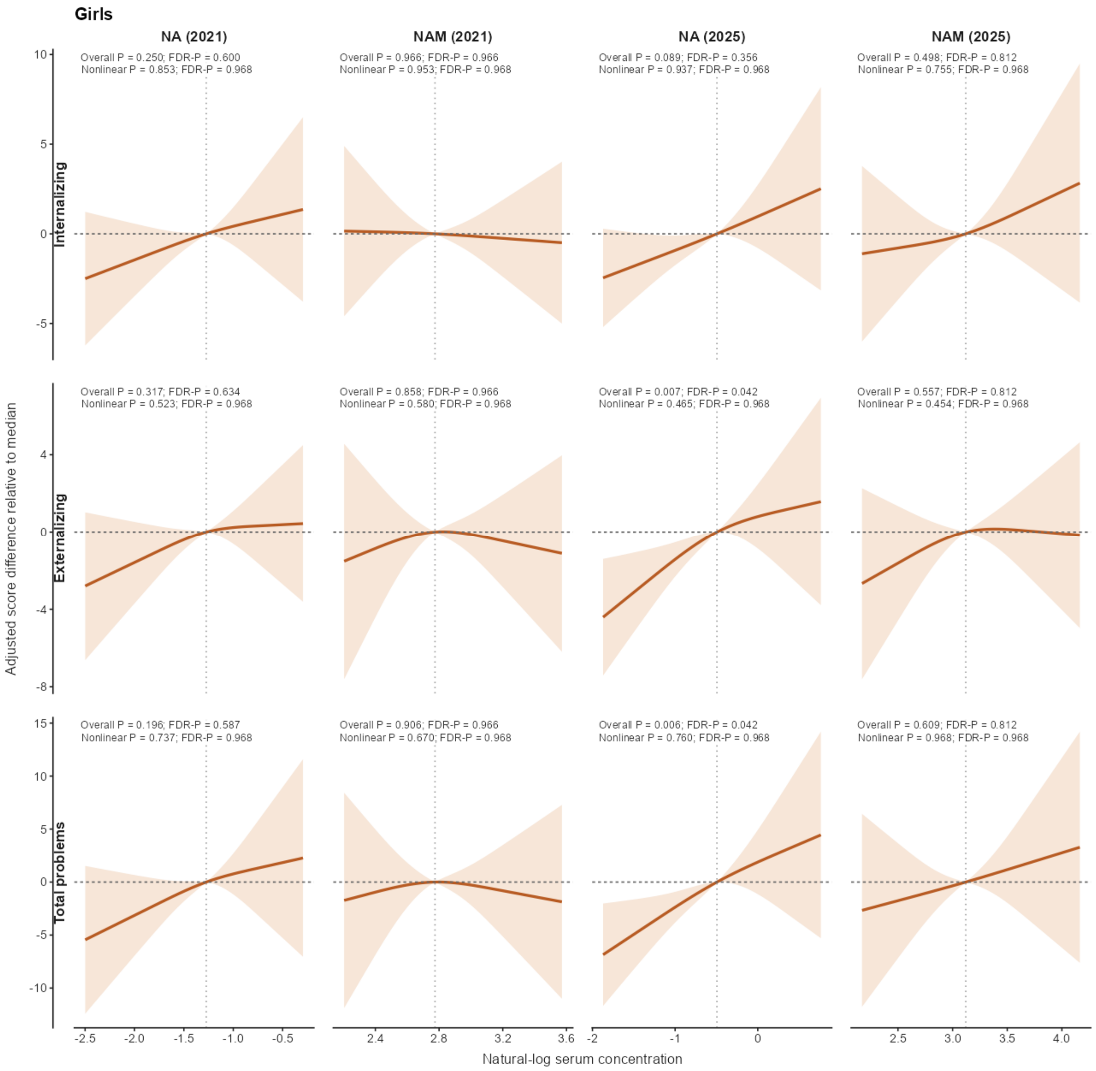

Figure S2. Restricted cubic spline associations of serum niacin and nicotinamide with CBCL outcomes for girls.
